# Mitochondrial reactive oxygen species as major effectors of antimicrobial immunity

**DOI:** 10.1371/journal.ppat.1008470

**Published:** 2020-05-28

**Authors:** Elena Shekhova

**Affiliations:** Medical Research Council Centre for Medical Mycology at the University of Exeter, University of Exeter, Exeter, United Kingdom; University of Massachusetts, Worcester, UNITED STATES

## Reactive oxygen species play an important function in innate immune cells

Reactive oxygen species (ROS) are key weapons against pathogenic bacteria and fungi in the antimicrobial defense arsenal of host immunity. Innate immune cells, namely macrophages and neutrophils, release ROS as cytotoxic effectors that can irreversibly oxidize and thus damage cellular structures of the intruding pathogens. At the same time, ROS are important intracellular mediators that drive the appropriate antimicrobial responses and tune the inflammatory response. The best-recognized source of ROS in phagocytic cells is the NADPH oxidase (NOX) complex [1]. However, mitochondria also contribute to the enhanced ROS generation in these cells. This review focuses on the underappreciated but important roles of mitochondrial ROS (mitoROS) in antimicrobial immune defenses.

## Mitochondria are one of two main sources of ROS in innate immune cells

ROS production in phagocytic cells is mainly mediated through the activity of the NOX complex. Upon pathogen recognition and engulfment, the NOX complex is formed within the phagosomal membranes, and it converts molecular oxygen into a highly reactive oxygen intermediate—superoxide [2]. Subsequently, other reactive intermediates can arise from NOX-derived superoxide depending on the pH levels, the presence of transitional metals, and other enzyme activities in activated phagocytes [3].

The mitochondrion is another cellular source of ROS in infected immune cells that is often overlooked. Interestingly, mitochondria produce low amounts of ROS even under normal, pathogen-free conditions. Superoxide can be generated at specific sites of the mitochondrial electron transport chain (ETC), for instance, at complex I or complex III. This may occur because of the escape of electrons from the electron carriers of the ETC to molecular oxygen [4–6].

Remarkably, the levels of mitoROS rise when phagocytes encounter microbes [7]. Studies on murine macrophages point towards a specific mechanism responsible for the increased mitoROS in infected cells. Upon macrophage activation, mitochondrial conditions favor reverse electron transport in the ETC. The infection-associated increase in the activity of the mitochondrial complex II likely leads to over-reduction of coenzyme Q, which is one of the electron carriers in the ETC. Consequently, electrons from coenzyme Q travel to one of the active sites of complex I, where, in turn, oxygen accepts electrons and forms superoxide [8,9]. Superoxide in mitochondria can be further converted into other ROS such as hydrogen peroxide (H_2_O_2_) in a reaction mediated by mitochondrial superoxide dismutase (Sod) [5]. Evidently mitochondria contribute, along with NOX, to the increased production of ROS in immune cells during infection. Although mitochondrial generation of ROS in infected immune cells has been well documented both in vitro and in vivo, the exact underlying mechanisms that activate mitoROS production remain poorly defined.

## Increased mitoROS production is induced specifically in infected immune cells

**Fig 1:**
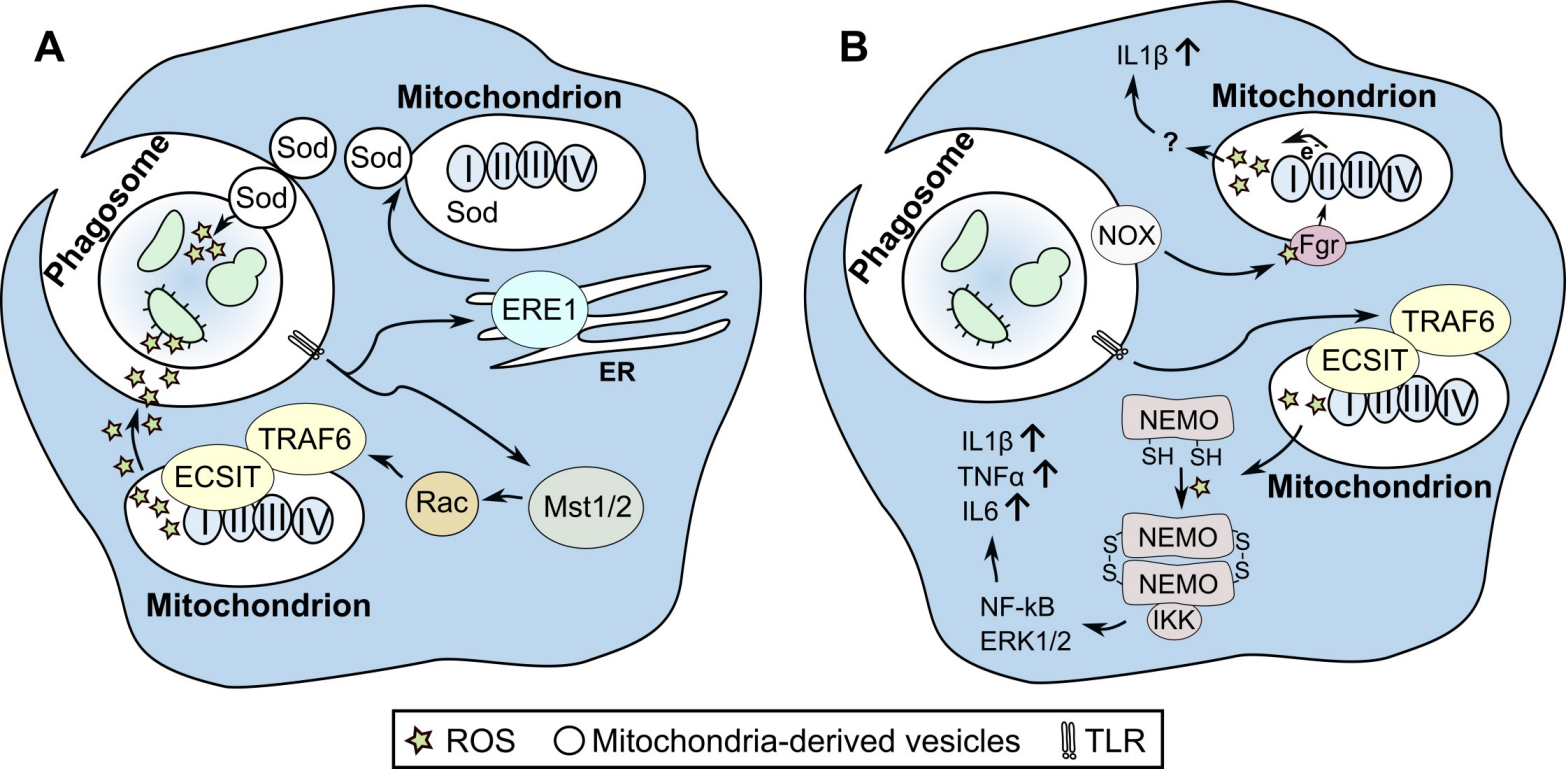
ROS contribute to the direct killing of microbes and regulate the production of proinflammatory cytokines. (A) TLR signaling increases the production of antibacterial mitoROS. MitoROS can reach the pathogen-containing phagosome because of the close proximity of mitochondria and phagosome. Juxtaposition of mitochondria and phagosome is regulated by the kinases Mst1 and Mst2, which act by activating small GTPase Rac. The activated Rac is required for translocation of the TLR signaling component TRAF6 to mitochondria [10]. Here, TRAF6 reacts with mitochondrial ECSIT, which is responsible for an assembly of the ETC complex I. The engagement of TRAF6 with mitochondrial ECSIT promotes the ubiquitination of the latter, which consequently augments mitoROS formation through disassembly of complex I of the ETC [11]. MitoROS can also reach phagosome through mitochondria-derived vesicles containing Sod [12]. TLRs activate ERE1α in the ER of infected phagocytes. Activated ERE1α promotes the formation of mitochondrial vesicles, which become accumulated inside the phagosome. These vesicles contain superoxide dismutase and thus contribute to mitoROS accumulation in the pathogen-containing phagosome [12]. (B) TLR signaling promotes inflammation through mitoROS. TLR signaling elevates generation of cytosolic ROS through the activity of NOX. Cytosolic ROS cause oxidation and a subsequent activation of redox-sensitive Src-type tyrosine kinase Fgr [13]. The activated Fgr increases the activity of mitochondrial complex II, which is required for the increase in mitoROS production via reverse electron transport in the ETC. MitoROS, in turn, may increase levels of proinflammatory cytokine IL1β, probably via inflammasome activation [8]. Independently of the activities of NOX, mitoROS can induce inflammation in response to invading pathogens. TLR signaling through TRAF6 can induce mitoROS generation in response to infection. Increased mitoROS levels induce oxidative modifications, in particular, intramolecular disulfide bonds, in NEMO. This redox modification of NEMO is required for binding and activating the IKK complex and leads to the activation of ERK1 and ERK2 and NF-κB pathways to increase the synthesis of proinflammatory cytokines IL1β, TNFα, and IL6 [18]. ECSIT, evolutionarily conserved signaling intermediate in Toll pathways; ERE1α, inositol-requiring enzyme 1α; ERK1/2, extracellular signal-regulated protein kinase 1/2; ETC, electron transport chain; Fgr, Gardner-Rasheed feline sarcoma viral (v-fgr) oncogene homolog; IKK, inhibitor of nuclear factor-κB (IκB) kinase; IL, interleukin; mitoROS, mitochondrial ROS; MST1/2, mammalian sterile 20-like kinases; NEMO, NF-κB essential modulator; NOX, NADPH oxidase; Rac, small guanosine triphosphate-binding protein; ROS, reactive oxygen species; Sod, superoxide dismutase; Src, proto-oncogene tyrosine-protein kinase; TLR, Toll-like receptor; TNFα, tumor necrosis factor α; TRAF6, tumor necrosis factor receptor-associated factor 6.

Sensing pathogens through pattern recognition receptors can trigger enhanced mitoROS production in immune cells. Once macrophages have recognized bacterial ligands via a subset of Toll-like receptors (TLRs) such as TLR1, TLR2, and TLR4, mitochondria are then recruited to the phagosomal membrane. The mammalian sterile 20-like kinases Mst1 and Mst2 are required for this juxtaposition of mitochondria and phagosome [10]. Meanwhile, the binding of tumor necrosis factor receptor-associated factor 6 (TRAF6) and a mitochondrial protein, evolutionarily conserved signaling intermediate in Toll pathways (ECSIT), promotes the increase in mitoROS production (Fig 1A) [11]. Interestingly, the TRAF6-ECSIT–dependent increase in mitoROS is required for oxidative killing of internalized *Salmonella typhimurium* by macrophages [11]. TLRs also influence the accumulation of mitoROS inside the phagosome via induction of mitochondria-derived vesicles. This happens when macrophages are challenged with *Staphylococcus aureus* [12]. In this scenario, endoplasmic reticulum (ER) stress induces the generation of mitochondrial vesicles containing Sod, which converts superoxide into H_2_O_2_ (Fig 1A) [12]. The functionality of TLR2/4/9 is required for these vesicles to accumulate inside the pathogen-containing phagosome, and this contributes to increased phagosomal concentrations of antibacterial H_2_O_2_.

TLR4 signaling is also linked to enhanced mitoROS generation, which in turn affects inflammation. This is observed in macrophages infected with *Escherichia coli*. Here, the increase in mitoROS is mediated by crosstalk between activated NOX and mitochondria (Fig 1B) [8]. Upon infection with live *E*. *coli*, NOX-derived ROS in macrophages react with the redox-sensitive Src-type tyrosine kinase, Fgr, which is activated in response to ROS exposure [8,13]. Fgr then increases the enzymatic activity of mitochondrial complex II, creating conditions for mitoROS production through reverse electron transport. The elevation of complex II activity, and thus the increase in mitoROS production, enhances accumulation of proinflammatory cytokine interleukin (IL)-1β, which in turn leads to the activation of the inflammatory program promoting bacterial killing [8]. This correlates well with the established notion that mitoROS positively regulate inflammasome formation and thus activate IL-1β [14].

Proinflammatory cytokines also increase mitoROS formation in infected immune cells. Cytokines such as interferon γ (IFNγ) and tumor necrosis factor (TNF) induce mitoROS production, which is essential for the elimination of *Listeria monocytogenes* and *Mycobacteria tuberculosis* [15–17]. However, the generation of mitoROS has to occur in a controlled manner to avoid host cell damage. Indeed, excessive TNF elevates levels of mitoROS and causes an overload of mitochondrial calcium in macrophages infected with *M*. *tuberculosis*. This ultimately results in the necrosis of macrophages and a release of bacteria to the extracellular environment, thereby exacerbating the infection [17].

Taken together, these findings show that mitoROS production can be induced specifically in infected immune cells. Triggering this system leads to the direct growth inhibition of the pathogen and the induction of inflammatory programs.

## Infection-associated redox reactions enable regulatory properties of ROS

The direct antimicrobial function of ROS in immune cells is mainly accomplished by creating oxidative stress and damaging cellular components of invading pathogens. As described above, signaling via mitoROS also contributes to antimicrobial immunity. However, little is known about the mechanisms behind the signaling properties of ROS, especially mitoROS, in infected immune cells. One possible mechanism is through the induction of oxidative modifications in regulatory proteins, which directly or indirectly mediate the synthesis of cytokines. For instance, in *L*. *monocytogenes*-infected macrophages, mitoROS are responsible for the formation of disulfide bridges in the NF-κB essential modulator (NEMO), which are required for dimerization of this protein (Fig 1B) [18]. Accordingly, the mitoROS-regulated formation of NEMO dimers induces a cascade of signaling reactions that subsequently leads to the secretion of proinflammatory cytokines, including IL-1β, TNFα, and IL-6 [18]. Therefore, mitoROS-mediated modifications of cellular regulators are involved in transforming macrophages into a proinflammatory state required to combat pathogens.

## Induction of mitoROS in immune cells is required to resist infection

ROS modulate the antimicrobial functions of innate immune cells by inhibiting the growth of invading pathogens as well as regulating inflammatory responses. Disruption of ROS-mediated processes leads to the inability of the host to clear the pathogens. A pathology such as chronic granulomatous disease, which is associated with the reduced production of ROS through NOX, is characterized by increased susceptibility to bacterial and fungal infections [19,20]. Another human immunodeficiency syndrome, which occurs because of a mutation in the gene encoding Rac2, is characterized by impaired NOX-derived and mitoROS production by phagocytic cells and associated with severe bacterial infections [10,21]. The importance of mitoROS for the host to clear microbes has been also proven in mouse models of infection. Mice that are deficient in proteins responsible for the induction of mitoROS are highly susceptible to infections caused by *S*. *typhimurium* and *L*. *monocytogenes* [11,16]. Similar to NOX-derived ROS [22], mitoROS also impact the rate of cytokine production during infection and thus play an essential role in regulating inflammation in vivo [8,9].

## Elevated levels of mitoROS may exhibit detrimental properties

Despite an essential role of mitoROS in antimicrobial responses of immune cells, mitoROS may also be responsible for damage of the host during infection. In particular, mitoROS may cause organ failure in several models of sepsis [23,24]. In corroboration, administration of specific inhibitors of mitoROS protect animals against organ damage in the lipopolysaccharide–peptidoglycan model of sepsis and lipopolysaccharide-induced endotoxemia [23,24] but do not exhibit a long-term beneficial effect in polymicrobial sepsis [25]. Moreover, mitoROS may contribute to exaggerated immune responses during viral infections such as infection with influenza A virus [26]. Accordingly, a pharmacological inhibitor of mitoROS, MitoTempo, can prevent lung inflammation and thus reduce mortality of mice infected with influenza A virus [26]. The harmful effects of mitoROS in these models might be associated with mitochondrial dysfunction, as well as impaired redox homeostasis. This possible correlation awaits further investigation.

## Future perspective

Multiple studies have proven the important role of mitoROS for antimicrobial immunity. To translate this knowledge into therapeutic opportunities, further mechanistic insights into the mode of action of mitoROS are needed. ROS act by reacting with various molecules such as DNA, proteins, or lipids. Functions or localization of these redox-sensitive molecules might be altered because of exposure to ROS. For instance, oxidative protein modifications may activate or inhibit protein functions. Thus, to fully understand the impact of the increased mitoROS levels in infected immune cells, cellular targets of mitoROS such as redox-sensitive proteins need to be defined. Also, mitoROS may alter permeability of mitochondrial membranes and thus act through releasing mitochondrial components into other cellular compartments. Indeed, in activated immune cells, mitoROS production leads to membrane permeability transition (MPT) and a subsequent release of mitochondrial DNA into the cytosol, where it increases concentrations of IL-1β [27]. The mechanisms behind the activation of MPT by mitoROS in the context of infection require further investigation.

As discussed here, mitoROS are important against pathogenic bacteria, but their function against pathogenic fungi is unknown. Thus, more studies are required to explore whether mitoROS also play an important role in antifungal immunity. Finally, besides being major effectors of the innate immunity, mitoROS also contribute to the induction and regulation of adaptive immune responses. For example, mitoROS play an essential role in the processes of antigen presentation by plasmacytoid dendritic cells [28]. Moreover, it is established that mitoROS levels affect T cell formation [29]. Currently, the exact molecular mechanisms by which mitoROS orchestrate adaptive immunity against pathogens remain largely unexplored.
